# Transcriptional response to an alternative diet on liver, muscle, and rumen of beef cattle

**DOI:** 10.1038/s41598-024-63619-2

**Published:** 2024-06-13

**Authors:** Anna Carolina Fernandes, Antonio Reverter, Kate Keogh, Pâmela Almeida Alexandre, Juliana Afonso, Julio Cesar Pascale Palhares, Tainã Figueiredo Cardoso, Jessica Moraes Malheiros, Jennifer Jessica Bruscadin, Priscila Silva Neubern de Oliveira, Gerson Barreto Mourão, Luciana Correia de Almeida Regitano, Luiz Lehmann Coutinho

**Affiliations:** 1 Department of Animal Science, Luiz de Queiroz College of Agriculture, University of São Paulo (ESALQ-USP), Piracicaba, São Paulo Brazil; 2 CSIRO Agriculture & Food, Queensland Bioscience Precinct, 306 Carmody Rd., St. Lucia, Brisbane, QLD 4067 Australia; 3 Animal and Bioscience Research Department, Teagasc, Animal & Grassland Research and Innovation Centre, Grange, Dunsany, Co. Meath Ireland; 4 Brazilian Agricultural Research Corporation, Embrapa Pecuária Sudeste, São Carlos, São Paulo Brazil; 5 Beef Cattle Research Center, Animal Science Institute (IZ), Sertãozinho, São Paulo Brazil; 6 Center of Biological and Health Sciences, Federal University of São Carlos, São Carlos, São Paulo Brazil

**Keywords:** Genomics, Data processing, Databases, Gene ontology, Gene regulatory networks, Genome informatics, Genetics, Functional genomics, Gene expression, Gene regulation, Genome, Genomics, Molecular biology, RNA metabolism, Transcription, Transcriptomics

## Abstract

Feed cost represents a major economic determinant within cattle production, amounting to an estimated 75% of the total variable costs. Consequently, comprehensive approaches such as optimizing feed utilization through alternative feed sources, alongside the selection of feed-efficient animals, are of great significance. Here, we investigate the effect of two diets, traditional corn-grain fed and alternative by-product based, on 14 phenotypes related to feed, methane emission and production efficiency and on multi-tissue transcriptomics data from liver, muscle, and rumen wall, derived from 52 Nellore bulls, 26 on each diet. To this end, diets were contrasted at the level of phenotype, gene expression, and gene-phenotype network connectivity. As regards the phenotypic level, at a *P* value < 0.05, significant differences were found in favour of the alternative diet for average daily weight gain at finishing, dry matter intake at finishing, methane emission, carcass yield and subcutaneous fat thickness at the rib-eye muscle area. In terms of the transcriptional level of the 14,776 genes expressed across the examined tissues, we found 487, 484, and 499 genes differentially expressed due to diet in liver, muscle, and rumen, respectively (*P* value < 0.01). To explore differentially connected phenotypes across both diet-based networks, we focused on the phenotypes with the largest change in average number of connections within diets and tissues, namely methane emission and carcass yield, highlighting, in particular, gene expression changes involving *SREBF2*, and revealing the largest differential connectivity in rumen and muscle, respectively. Similarly, from examination of differentially connected genes across diets, the top-ranked most differentially connected regulators within each tissue were *MEOX1*, *PTTG1,* and *BASP1* in liver, muscle, and rumen, respectively. Changes in gene co-expression patterns suggest activation or suppression of specific biological processes and pathways in response to dietary interventions, consequently impacting the phenotype. The identification of genes that respond differently to diets and their associated phenotypic effects serves as a crucial stepping stone for further investigations, aiming to build upon our discoveries. Ultimately, such advancements hold the promise of improving animal welfare, productivity, and sustainability in livestock farming.

## Introduction

The feed cost is a critical economic factor in cattle production, accounting for approximately 75% of the total variable costs involved in beef production systems^1^. The dominant position of cost of feed in the economic landscape of cattle production underscores the importance of managing feed resources effectively. As feed costs directly impact profitability, fluctuations in feed prices or inefficiencies in feed utilization can significantly influence beef production sustainability. Thus, multifaceted comprehensive strategies including, but not limited to, incorporation of alternative feeds, coupled with optimization of feed utilization through genetic improvement for feed efficiency and leveraging genomic tools, emerge as crucial approaches to mitigate the financial burden imposed by feed expenses, with added benefits of minimizing the environmental footprint and enhancing the sustainability of the production system.

The incorporation of cost-effective feed alternatives to traditional grain-based diets is a promising avenue to tackle the high inputs of feed cost in cattle production^2,3^. For instance, crop residues and industrial by-products can contribute to reducing dependence on expensive grains, as well as the environmental footprint of cattle farming operations, leading to reduced pressure on natural resources and lowered greenhouse gas emissions, such as methane, therefore enhancing overall efficiency and sustainability of livestock production.

Methane is a potent greenhouse gas, estimated to have 28 times greater warming potential than carbon dioxide^4^. Approximately 2–12% of the total energy consumed by ruminants is released as emitted methane during digestion^5^, which represents a natural loss of energy that could have otherwise been harnessed to enhance animal productivity. In that sense, reducing enteric methane emissions from cattle holds potential to not only align with global efforts to combat climate change and promote more eco-friendly livestock management but also improve animal performance. Consequently, adopting alternative diets can assist in a more resilient and ecologically responsible livestock industry, supporting long-term economic viability and environmental conservation, as well as fortify long-term agricultural sustainability, contributing to a more environmentally friendly and ethical approach to meat production. It can also decrease the competition for grains (e.g. corn and soybean) with human nutrition^6^.

Furthermore, the implementation of breeding selection programs to identify and subsequently breed genetically superior individuals able to efficiently use alternative feeds with optimal feed conversion rates is a key approach. Feed-efficient animals consume less food to produce the same amount of meat^7^, and emit less methane per unit of output^8,9^, thereby leading to increased system productivity and profitability, and reduced resource usage and environmental footprint. Moreover, utilizing high-throughput genomic technologies, such as genome-wide association studies (GWAS) and genomic selection (GS), enables the identification of genetic markers associated with feed, methane emission and production efficiency traits^10–14^. These markers are powerful tools for predicting genetic potential^15^, allowing for more informed breeding decisions, ultimately expediting genetic progress, and enabling faster dissemination of superior genetic traits across cattle populations.

While lagging behind humans and model organisms, there is a growing number of studies in livestock species regarding the impact of dietary components on gene expression patterns^16^, ultimately leading to phenotype modulation. However, it is still unclear whether these differences can be attributed to the coordinated function of genes and pathways regulation. The genetic architecture behind complex traits involves a variety of genes that are sensitive to environmental changes^17^, and, in turn, determining the mechanisms responsible for these changes is equally challenging.

In the present study, our objective was to investigate the effect of two competing diets on economically relevant phenotypes related to feed, methane emission and production efficiency and on multi-tissue transcriptomics data from three major metabolic tissues—liver, muscle, and rumen wall—derived from Nellore bulls. The application of gene co-expression network analyses may shed light on a comprehensive and systems-level view of dietary responses at the molecular level, uncovering regulatory networks and biological pathways impacting production traits through differential gene regulation^18–20^. By gaining greater insight into the intricate relationships between genes and their coordinated responses to dietary inputs, this knowledge can contribute to advancing our understanding of the molecular mechanisms that are central to phenotype determination in livestock, and eventually assist in the development of precision nutrition strategies to optimize livestock performance and sustainability.

## Methods

### Overview

**Figure 1:**
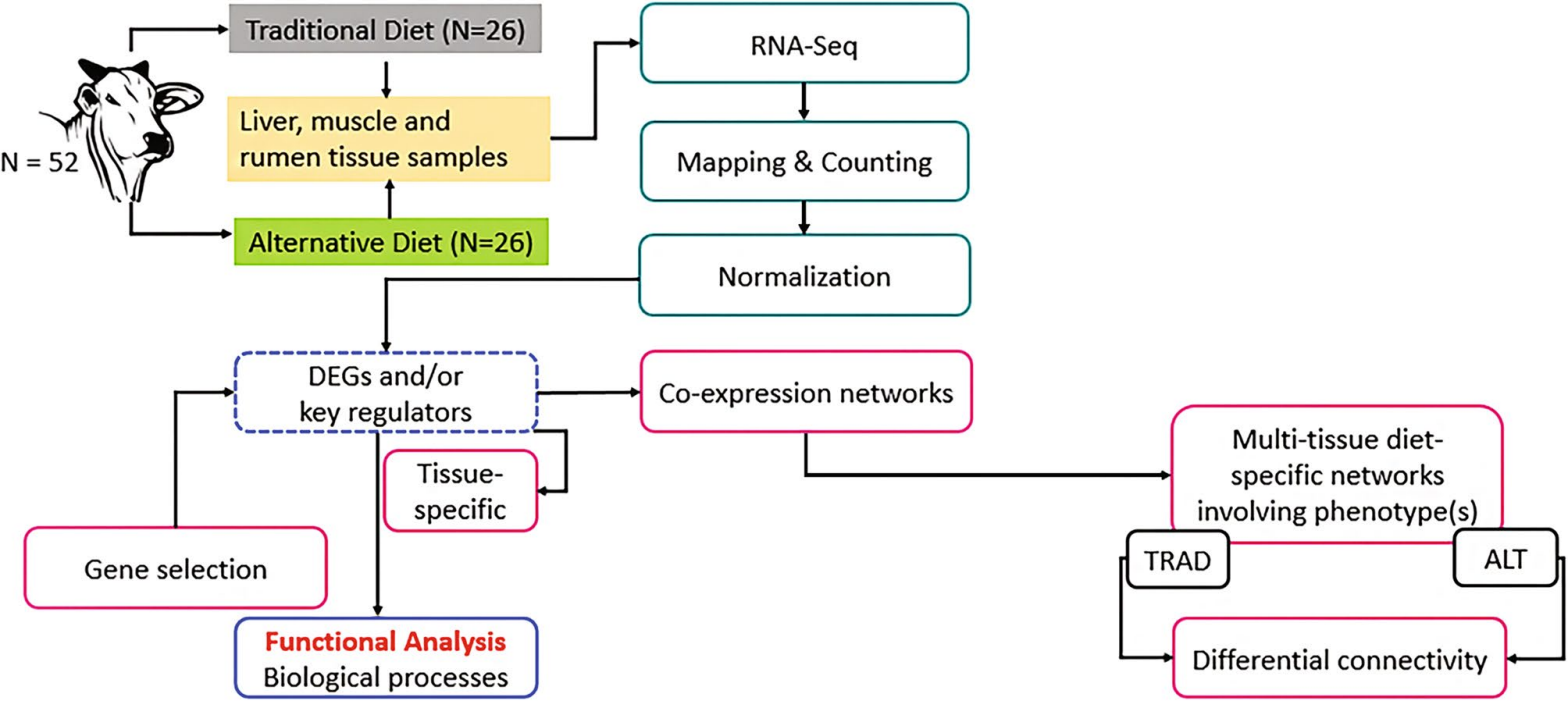
Bioinformatic workflow of the multi-tissue RNA-seq-based gene co-expression networks. *DEGs* differentially expressed genes, *TRAD* traditional diet, *ALT* alternative diet.

An overview of the methodological workflow for the current study, from group formation and tissue sampling up to gene co-expression analysis and differential connectivity is shown in Fig. 1, and specific details of each component are given in the forthcoming paragraphs.

### Animal resource, tissue, and phenotype collection

All experimental procedures were conducted in accordance with animal welfare and humane slaughter guidelines and were approved by the EMBRAPA Livestock Science Ethics Committee on Animal Experimentation, São Carlos, São Paulo, Brazil (Protocol No. 09/2016). All methods were performed in accordance with relevant guidelines and regulations. Methods are reported in the manuscript following the recommendations in the ARRIVE guidelines.

We used a population of 52 Nellore bulls (*Bos taurus indicus*) born in 2014, derived from an experimental herd of the Brazilian Agricultural Research Corporation (EMBRAPA), and sired by 28 unrelated commercial bulls, contributing an average of 1.85 progenies, and ranging from 1 to 5 progenies. Between December 2014 and July 2016, the animals were raised in grazing pastures. From July to November 2016 (121 days), the experiment was conducted at the feedlot facility belonging to EMBRAPA Southeast Livestock, located in São Carlos (São Paulo, Brazil), with cattle divided into two subgroups by initial body weight (light or heavy), with light and heavy animals evenly allocated within each group. The experimental feedlot was composed of four pens, equipped with automated feeding systems (Model 6000, GrowSafe^®^ Systems Ltd., Airdrie, Alberta, Canada), capable of registering live weight and daily food consumption for each individual animal.

The study included two dietary treatments (Table 1), as follows: a high-grain traditional diet (TRAD) based on corn grains and soybean meal (n = 26 bulls) and a by-product alternative diet (ALT) based on corn germ oil meal, citrus pulp and peanut shell meal (n = 26). In both treatments, all animals received urea, mineral supplements, active dry yeast, virginiamycin, and monensin. The total mixture composed of corn silage and concentrates was provided twice a day (9 a.m. and 4 p.m.) and adjusted daily to ensure that the animals had ad libitum access to food.

**Table 1:** Description of diets: ingredients and nutritional composition of the traditional and alternative diets.

Traditional diet		Alternative diet	
Ingredients, % of DM			
Corn silage	46.6	Corn silage	30.0
Corn grains	41.6	Corn germ oil meal	35.9
Soybean meal	6.0	Citrus pulp	24.0
Protected fat	2.5	Peanut shell meal	7.5
CONFINATTO N235 2^a^	2.0	CONFINATTO N235 2	2.1
Urea	1.3	Urea	0.5
Predicted nutrient composition, DM basis			
Intake, kg DM/day	11.40	Intake kg, DM/day	10.70
Total digestible nutrients (TDN), %	74.86	Total digestible nutrients (TDN), %	73.19
Crude protein (CP), %	13.91	Crude protein (CP), %	14.81
Fat, %	5.40	Fat, %	6.62
Calcium, %	0.66	Calcium, %	0.94
Phosphorus, %	0.31	Phosphorus, %	0.53

^a^CONFINATTO N235 2: mineral supplements + active dry yeast + virginiamycin + monensin.

After a dietary adaptation period of 10 days, body weight (BW, kg) of all animals was recorded at three-time points, each following a 16-h fasting period: feedlot entry (July), intermediate feedlot stage (September) and feedlot exit (November). Individual dry matter intake (DMI, kg/day) and average daily gain (ADG, kg/day) were measured at both feedlot phases: growing (backgrounding) and finishing (fattening). DMI was obtained by the difference between the weight of the diet provided and refusal, and ADG was estimated by the difference between the end and entry weight divided by the number of days in feedlot. Methane emission (ME, g/day) was measured during the finishing phase of feedlot, using the GreenFeed system (Clock Inc., Rapid City, SD, United States), which consists of an automated head-chamber device that uses sensors to estimate daily methane emission from individual cattle by measuring gas concentrations and airflow over 3–7 min, multiple times a day, during several days (further details in Huhtanen et al.^21^ and Hristov et al.^22^). In brief, the GreenFeed system was gradually introduced to the animals without required immediate interaction with it, allowing them to investigate and become accustomed to its presence over time. Then, we used a positive reinforcement technique to encourage them to approach and interact with it, rewarding them with treats when they engaged with the system or showed interest in it. The equipment was programmed to provide feed pellets to attract five daily visits per animal.

At the end of the experimental period, all animals were transported for slaughter at a commercial slaughterhouse located in Bariri (São Paulo, Brazil), under Federal Inspection Service supervision and Brazilian Ministry of Agriculture, Livestock and Food Supply legislation (Normative Instruction 03/2000). The animals were slaughtered at an average age of 23.5 months, using the pneumatic pistol stunning technique and cutting of the jugular vein, followed by removal of the hide, head, feet, and evisceration.

At slaughter, samples from liver, *Longissimus dorsi* (LD) muscle, and rumen wall from all 52 animals were collected. The liver and muscle biopsies were harvested from the median portion of the left liver lobe and between the 12th and 13th ribs, respectively. As for the rumen samples, a 2–3 cm^2^ piece of bulk tissue was collected from the rumen wall, including all rumen wall layers (the stratified epithelium, surrounded by a muscular layer and the submucosa). To standardize the collection position across samples, we used the esophagus opening and reticulum as references. The sampling site in the lateral rumen wall was defined at approximately 5 cm below the esophagus and immediately after the border of the reticulum. All samples were immediately snap frozen in liquid nitrogen to preserve RNA integrity, and stored at a − 80 °C freezer until total RNA extraction. For all tissues, care was provided to ensure that samples were consistently taken from the same location from each animal.

The carcasses were weighed to obtain the hot carcass weight (HCW, kg) before being transferred to cold rooms. From this value, as well as the slaughter live body weight, the carcass yield (CY, %) was calculated. Then, the carcasses were split longitudinally with a band saw to obtain the left and right halves. After cooling at 4 °C for a 24-h period, a cross-sectional cut was made on the left half side of the carcasses, exposing the LD muscle between the 12th and 13th ribs, to measure the rib-eye muscle area (REA, cm^2^) using a checkered grid. Measure of the subcutaneous fat thickness at the REA (FT_REA, mm), using a digital caliper ruler, was performed over the same cross-section, at a point three-fourths of the length of the LD muscle from the split chine bone.

### Statistical analysis of phenotypes

A total of 14 phenotypes were collected: three estimates of average daily weight gain relative to growing (ADG_G), finishing (ADG_F) and growing + finishing (ADG_GF) feedlot phases; three body weight measurements taken at the beginning (BW1), mid-point (BW2) and end of the experiment (BW3); three dry matter intake estimates relative to growing (DMI_G), finishing (DMI_F) and growing + finishing (DMI_GF) feedlot phases; methane emission (ME), hot carcass weight (HCW), carcass yield (CY), rib-eye muscle area (REA) and subcutaneous fat thickness at the rib-eye muscle area (FT_REA).

The statistical analyses were aimed at testing the effect of diet on phenotypic variation. These were performed using the General Linear Model procedure of the SAS statistical software (PROC GLM; SAS Institute Inc., Cary, NC, USA). In the analysis of variance (ANOVA) model, age, diet treatment, and interaction between weight group × pen (nested within diet) were used as independent variables. Diet least-squared means (LSM) and standard errors (SE) were estimated, and differences declared statistically significant if t-test P value < 0.05.

### RNA extraction, cDNA library preparation, and transcriptome sequencing

Total RNA was extracted from the liver, muscle and rumen wall tissue samples using the TRIzol reagent (Thermo Fisher Scientific, Waltham, MA, USA), according to the manufacturer's instructions. The NanoDrop 2000 Spectrophotometer (NanoDrop Technologies, Wilmington, DE, USA) and Qubit^®^ 2.0 Fluorometer were employed to quantify the total RNA concentration and quality, while the RNA integrity was evaluated by using the Agilent Bioanalyzer 2100 (Agilent, Santa Clara, CA, USA). All samples presented an RNA integrity number (RIN) greater than or equal to 7 and were subjected to cDNA synthesis.

For cDNA library preparation, 2 μg of total RNA from each tissue sample were used, according to the protocol described in the Illumina Stranded mRNA Prep Ligation reference guide (Illumina, San Diego, CA, USA). The average library size was estimated using the Agilent Bioanalyzer 2100, and libraries were quantified using a KAPA qPCR Library Quantification Kit (KAPA Biosystems, Foster City, CA, USA).

The sequencing was carried out on the Illumina NextSeq2000 platform (Illumina, San Diego, CA, USA), generating paired-end reads (100 bp) at a depth of 40–50 M reads per sample, following standard protocols. All the sequencing analyses were performed at the ESALQ-USP Functional Genomics Center at the Animal Biotechnology Laboratory of ESALQ-USP, Piracicaba, São Paulo, Brazil.

### RNA-seq data analysis, differentially expressed genes (DEGs) and false discovery rate (FDR)

The RNA-seq reads were quality-checked, trimmed, and aligned to the ARS-UCD1.2 bovine reference genome^23^ using the Nextflow v22.10.1^24^ RNA-seq pipeline, nf-core/rnaseq v3.10.1^25^. In brief, raw read quality control was assessed using FastQC v0.12.0 (http://www.bioinformatics.babraham.ac.uk/projects/fastqc/), adapters and any low-quality reads were removed with Trim Galore! v0.6.7 (https://github.com/FelixKrueger/TrimGalore)^26^, trimmed FastQ files were mapped to the bovine reference genome (ARS-UCD1.2) using STAR v2.7.10a^27^ and quantification (raw counts per gene) was performed using RSEM v1.3.1^28^. All tool versions and full details of the pipeline are available at https://nf-co.re/rnaseq.

The gene expression normalization was performed across all samples and within the three tissues together using edgeR v.3.36.0^29^ in R v.4.1.3^30^. The raw read counts were transformed to log2 counts per million (CPM), lowly expressed genes (CPM < 1 in 50% of the samples) were filtered out, and libraries were normalized by the trimmed mean of M-values (TMM) approach^29^. After normalization, the genes which presented at least 1 CPM in at least half of the samples were retained for differential expression, subsequently performed within tissue between two diets by Student's t test with *P* value < 0.01 considered statistically significant.

Following Bolormaa et al.^31^ in the context of genome-wide association studies and with equivalent original derivations from Storey^32^, False Discovery Rate (FDR) was calculated as:

$$\text{FDR}= \frac{P\left(1-\frac{{N}_{DE}}{T}\right)}{\left(\frac{{N}_{DE}}{T}\right)\left(1-P\right)}$$

where *P* is the *P* value tested, *N*_*DE*_ is the number of differentially expressed genes that were significant at the *P* value tested, and *T* is the total number of genes tested (*T* = 14,776).

### Identification of key transcription factors (TFs) and cofactors (COFs)

To detect candidate TFs and COFs differentially regulating gene expression in the TRAD and ALT diets in each tissue, we employed the Regulatory Impact Factor (RIF) metrics, namely RIF1 and RIF2^33^. These algorithms operate under the assumption that key regulators within a network alter their behaviour in distinct biological conditions (i.e., diet treatment in our case), thereby contributing to differential gene expression. The RIF1 score identifies regulators that exhibit differential connectivity with the target genes, while RIF2 assesses TFs or COFs with potential to serve as predictors of target gene abundance. To identify such regulators, a list of known bovine TFs and COFs, obtained from the Animal Transcription Factor Database 4.0^34^, was compared to the list of DEGs for each diet in each tissue. To assign a significance level, each score was transformed into a *Z*-score, and values located ± 1.96 standard deviation from the mean, corresponding to *P* value < 0.05, were considered significant.

### Functional enrichment and visualization

The resultant list of DEGs, TFs and COFs were subjected to functional overrepresentation analysis to determine over or under-represented biological processes underpinning relevant functions using PANTHER v.17.0 (http://www.pantherdb.org/)^35^, considering a FDR ≤ 0.05 as threshold. Additionally, a hierarchical clustering analysis was performed using PermutMatrix v.1.9.4 (http://www.atgc-montpellier.fr/permutmatrix/)^36^. The row-by-row normalization was performed on the data using the *Z*-score transformation. Euclidean distance method was used for data aggregation. Venn diagrams were obtained using the VennDiagram v.1.7.3^37^ package in R v.4.1.3^30^.

### Gene networks

For the gene co-expression networks inference, all the genes identified as DEGs, key TFs or COFs, and the 14 phenotypes, considering all animals and tissues together, were used as nodes, and the significant connections between them were identified by using the Partial Correlation and Information Theory (PCIT) algorithm^38^. This approach aims to determine the significance of the correlation between a pair of nodes after accounting for all other nodes within the network. Initially, to identify changes in the gene network topology between diets, we built diet-specific networks across all tissues. Secondly, significantly correlated pairs were selected when at least one phenotype was present. In addition, we also identified significant connections unique or shared across the competing diets, creating a network with the shared connections. The resultant networks were imported into Cytoscape v3.10.0^39^ for visualization.

### Differential connectivity

Finally, considering each diet-specific network, we focused on exploring differentially connected phenotypes across both networks, to identify changes in behaviour depending on the biological condition (diet), moving from highly connected to lowly connected and vice-versa. The two networks, one considering each diet, were contrasted by focusing on the phenotypes with the largest change in the average number of connections within diets and tissues. The resultant sub-networks from each diet-specific network were extracted and visualized using Cytoscape^39^.

## Results

### Phenotypic data

In this study, we analyzed 14 traits: ADG_G, ADG_F, ADG_GF, BW1, BW2, BW3, DMI_G, DMI_F, DMI_GF, ME, HCW, CY, REA and FT_REA. Summary statistics for all phenotypes are outlined in Table 2.

**Table 2:** Descriptive statistics from phenotypic values for traits analysed in the current study (N = 52 cattle).

Phenotypes^a^	Mean	SD^b^	Minimum	Maximum
ADG_G, kg/day	1.74	0.33	0.86	2.43
ADG_F, kg/day	1.49	0.41	0.40	2.26
ADG_GF, kg/day	1.61	0.25	0.92	2.15
BW1, kg	325.75	34.60	255.00	391.00
BW2, kg	452.18	41.24	364.00	524.00
BW3, kg	478.18	39.74	370.50	555.00
DMI_G, kg/day	7.20	1.99	2.62	11.96
DMI_F, kg/day	8.89	1.89	5.80	14.88
DMI_GF, kg/day	8.04	1.69	5.11	13.12
ME, g/day	170.06	27.67	88.52	223.58
HCW, kg	263.69	23.52	205.50	311.50
CY, %	55.14	1.50	52.11	58.79
REA, cm^2^	69.48	7.99	52.25	86.50
FT_REA, mm	4.15	1.50	2.00	9.00

^a^ADG_G: average daily weight gain estimate relative to the growing phase in kg/day; ADG_F: average daily weight gain estimate relative to the finishing phase in kg/day; ADG_GF: average daily weight gain estimate relative to the growing + finishing phases in kg/day: BW1: body weight taken at the beginning of the experiment in kg; BW2: body weight taken at the mid-point of the experiment in kg; BW3: body weight taken at the end of the experiment in kg; DMI_G: dry matter intake estimate relative to the growing phase in kg/d; DMI_F: dry matter intake estimate relative to the finishing phase in kg/day; DMI_GF: dry matter intake estimate relative to the growing + finishing phases in kg/day; ME: methane emission in grams/d; HCW: hot carcass weight in kg; CY: carcass yield in %; REA: rib-eye muscle area in cm^2^; FT_REA: fat thickness at the rib-eye muscle area in mm.

^b^Standard deviation of the population.

On average, the ANOVA model for the analysis of the phenotypes accounted for 30% of the variance, ranging from 5.32% for DMI_G to 62.14% for BW1 (Table 3). Also presented in Table 3 is the effect of diet on each phenotype. At a nominal *P* value < 0.05 there were no statistically significant differences for nine of the 14 phenotypes: ADG_G, ADG_GF, BW1, BW2, BW3, DMI_G, DMI_GF, HCW and REA. However, significant differences were found for ADG_F, DMI_F, ME, CY, and FT_REA. These include an effect of the alternative diet resulting in a 12.9% higher growth at finishing, 22.1% higher dry matter intake at finishing, 10.9% lower methane emission, 1.7% higher carcass yield, and 31.5% higher subcutaneous fat thickness at the rib-eye muscle area.

**Table 3:** Percentage of variation (R^2^), least-square means, and *P*-value for the test of differences between diets.

Phenotypes^a^	R^2^, %	LSMean ± SE^b^		*P* value
		Traditional diet	Alternative diet	
ADG_G, kg/day	11.23	1.78 ± 0.06	1.71 ± 0.06	0.4747
ADG_F, kg/day	51.87	1.40 ± 0.06	1.59 ± 0.06	0.0255
ADG_GF, kg/day	27.09	1.56 ± 0.04	1.65 ± 0.04	0.1226
BW1, kg	62.14	328.96 ± 4.36	322.54 ± 4.36	0.3038
BW2, kg	46.07	451.71 ± 6.20	452.66 ± 6.20	0.9142
BW3, kg	27.65	476.94 ± 6.91	479.43 ± 6.91	0.7999
DMI_G, kg/day	5.32	7.65 ± 0.40	6.75 ± 0.40	0.1149
DMI_F, kg/day	36.45	8.00 ± 0.31	9.77 ± 0.31	0.0002
DMI_GF, kg/day	8.74	7.84 ± 0.33	8.24 ± 0.33	0.3940
ME, g/day	31.09	178.86 ± 4.70	161.26 ± 4.70	0.0110
HCW, kg	28.23	260.62 ± 4.07	266.77 ± 4.07	0.2918
CY, %	27.01	54.67 ± 0.26	55.61 ± 0.26	0.0145
REA, cm^2^	25.71	68.29 ± 1.41	70.67 ± 1.41	0.2369
FT_REA, mm	17.39	3.59 ± 0.28	4.72 ± 0.28	0.0063

^a^For definition of phenotype abbreviation, see footnote on Table 2.

^b^*LSMean* least-squared means, *SE* standard error.

### RNA-seq data analysis

We obtained 7.02 billion clean reads from 153 RNA-seq samples including 52 from liver, 50 from muscle, and 51 from rumen tissue. An average of 48.6, 51.1 and 37.2 million reads were obtained from each tissue, respectively. After quality control, 90.4%, 90.8%, and 83.5% of unique reads, on average, from liver, muscle, and rumen, respectively, were mapped to the reference genome (Table 4). The full table with alignment statistics per sample and tissue is reported in Supplementary Files S1, S2 and S3 for liver, muscle, and rumen, respectively.

**Table 4:** Summary of RNA-sequencing throughput and mapping rates per tissue.

Tissue	M Total seqs^a^	% Mapped^b^	M Reads mapped^c^	% GC^d^	Error rate^e^
Liver	48.6	90.4	43.9	48	0.37
Muscle	51.1	90.8	46.4	52	0.33
Rumen	37.2	83.5	31.8	49	0.35

^a^M Total seqs: average total sequences in the bam file (millions).

^b^Mapped: average % of mapped reads.

^c^M Reads Mapped: average reads mapped in the bam file (millions).

^d^GC: average % GC content.

^e^Error rate: average mismatches/base mapped.

### Differential expression analysis

Under the expression threshold of CPM ≥ 1 in at least 50% of samples (multi-tissue normalization approach, see “Methods”), 14,776 genes (53.5%) out of 27,607 reported on the Ensembl annotation file, were expressed in liver, muscle, and rumen tissues. Table 5 shows the number of DEGs that would have been captured at various *P* value thresholds and the corresponding FDR. As expected, the number of DEGs and FDR decreases with increasing significance (i.e. decreasing *P* value thresholds). At the nominal *P* value of < 0.01 the FDR was ~ 30% for each of the tissues. While this FDR value could be considered high, similar FDR has been reported in the context of GWAS (see for instance Table 2 in Reverter et al.^40^) and this threshold gave us a sizable number of genes of ~ 500 from each tissue, the relevance of which will be revealed in the forthcoming network-based analyses.

**Table 5:** Number of significant differentially expressed genes and FDR at decreasing *P* value thresholds for each tissue.

*P* value	Liver		Muscle		Rumen	
	N_DE_^a^	FDR^b^, %	N_DE_	FDR, %	N_DE_	FDR, %
< 0.05	1252	56.9	1261	56.4	1151	62.3
< 0.01	487	29.6	484	29.8	499	28.9
< 0.005	349	20.8	350	20.7	385	18.8
< 0.001	190	7.7	180	8.1	211	6.9
< 0.0005	146	5.0	139	5.3	181	4.0
< 0.0001	85	1.7	81	1.8	125	1.2
< 0.00005	68	1.1	72	1.0	106	0.7
< 0.00001	45	0.3	48	0.3	78	0.2

^a^*N*_*DE*_ number of significant differentially expressed genes.

^b^*FDR* false discovery rate.

**Figure 2:**
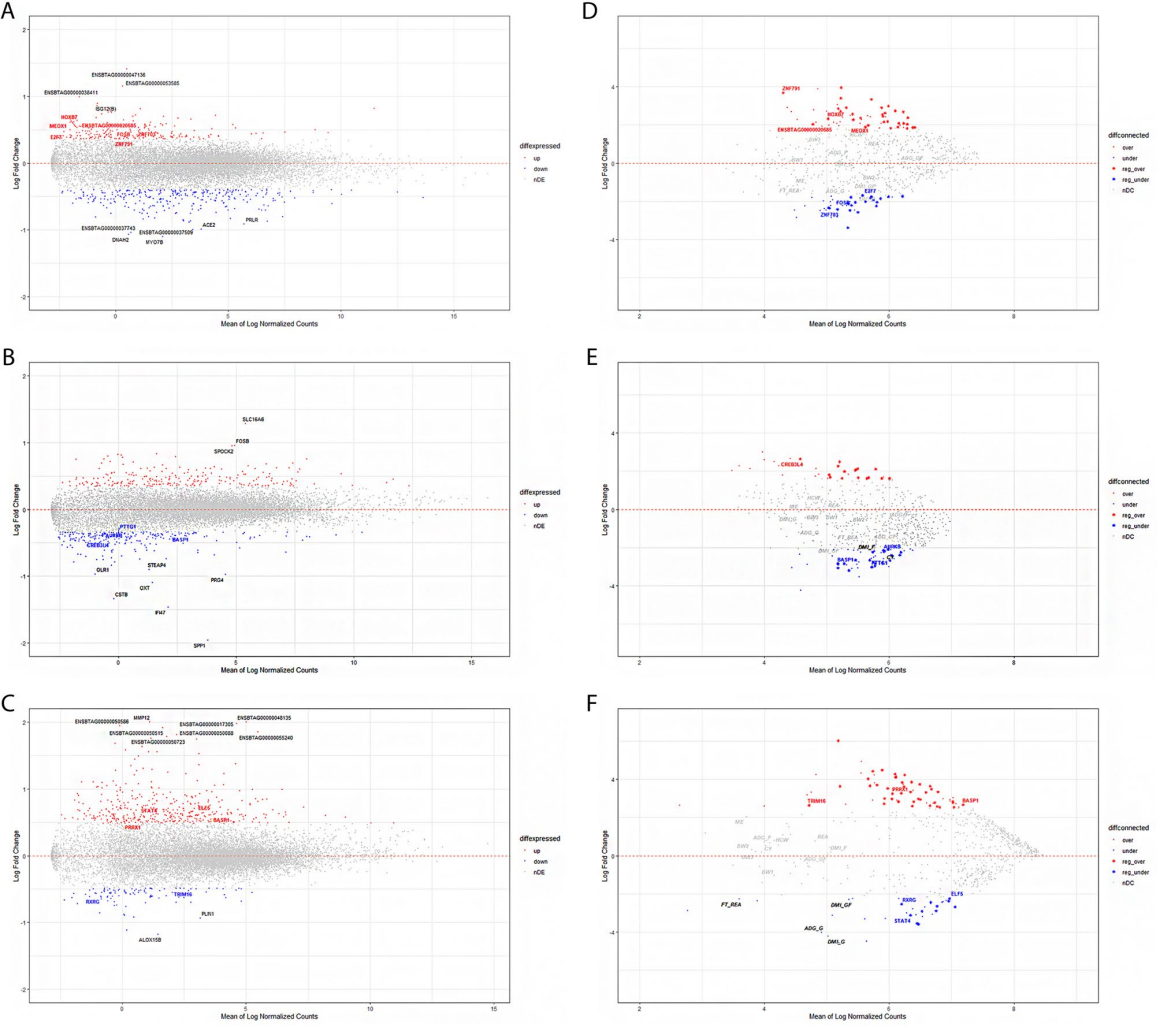
MA-Plots for expression (left panels) and connectivity (right panels) comparing the difference (Log Fold Change, y-axis) against the mean (Mean of Log Normalized Counts, x-axis) for liver (**A**,**D**), muscle (**B**,**E**) and rumen (**C**,**F**). Highlighted in red (blue) are the differentially over-expressed (under-expressed) or over-connected (under-connected) genes. Location of relevant genes and phenotypes is also given with top-ten up or down in black font.

Therefore, we retained a total of 487, 484, and 499 genes for liver, muscle, and rumen, respectively, identified as DEGs (*P* value < 0.01) (Fig. 2). The full list of DEGs at a *P* value < 0.01 for each tissue is presented in Supplementary Files S4, S5 and S6 for liver, muscle, and rumen, respectively.

From the 487 DEGs in liver, 194 genes were up-regulated (log2FoldChange ranging from + 0.36 to + 1.41) and 293 genes were down-regulated (log2FoldChange ranging from − 1.10 to − 0.39). The genes with the most altered expression were ENSBTAG00000047136 (log2FoldChange =  + 1.41; *q*-value = 7.63 × 10^–19^) and *MYO7B* (log2FoldChange = − 1.10; *q*-value = 1.18 × 10^–11^). The ENSBTAG00000047136 gene has been previously found within a genomic region on the bovine chromosome 14, associated with carcass-related metrics in dairy and beef sires^41^. *MYO7B* was identified within imprinted lead SNPs of QTL regions for carcass conformation in cattle^42^.

Regarding muscle, within the 484 DEGs, 234 were up-regulated (log2FoldChange ranging from + 0.34 to + 1.29) and 250 were down-regulated (log2FoldChange ranging from − 1.96 to − 0.34). The genes with the most altered expression were *SLC16A6* (log2FoldChange =  + 1.29; *q*-value = 1.18 × 10^–18^) and *SPP1* (log2FoldChange = − 1.96; *q*-value = 5.86 × 10^–41^). *SLC16A6* is a common DEG reported to harbour QTLs related to carcass and meat quality traits^43^, whereas *SPP1* is a candidate gene influencing carcass traits^44^.

Finally for rumen, among the 499 DEGs, 397 were up-regulated (log2FoldChange ranging from + 0.48 to + 2.06) and 102 were down-regulated (log2FoldChange ranging from − 1.18 to − 0.49). The genes with the most altered expression were ENSBTAG00000048135 (log2FoldChange =  + 2.06; *q*-value = 1.70 × 10^–23^) and *ALOX15B* (log2FoldChange = − 1.18; *q*-value = 7.93 × 10^–9^). ENSBTAG00000048135, an unannotated gene with unknown function in cattle, is a homologue of human *IGHG* gene^45^, known to influence the innate immune function of IgG molecules and B-cells^46^. The *ALOX15B* gene has been previously identified as a down-regulated DEG within the inflammatory response function in feedlot-crossbred cattle^47^.

**Figure 3:**
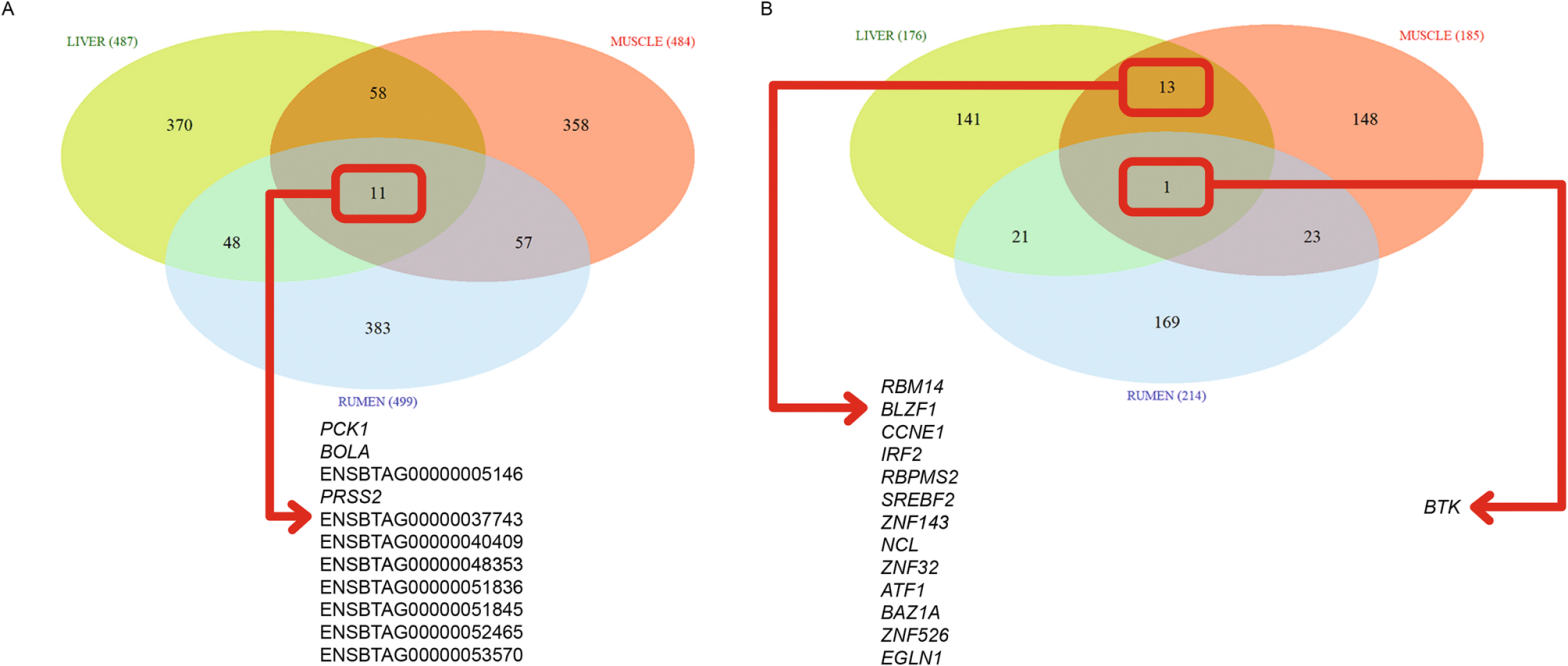
Venn diagram of differentially expressed genes (left) and key transcription factors and cofactors (right) identified across liver, muscle, and rumen tissue samples for the contrast between TRAD and ALT diets.

Of the genes designated as differentially expressed within this study, 11 were commonly reported as differentially expressed across the three tissues. Within the shared genes, 3 were known-genes—*BOLA*, *PCK1*, *PRSS2*—whereas 8 were unannotated—ENSBTAG00000005146, ENSBTAG00000037743, ENSBTAG00000040409, ENSBTAG00000048353, ENSBTAG00000051836, ENSBTAG00000051845, ENSBTAG00000052465 and ENSBTAG00000053570 (Fig. 3). The bovine leukocyte antigen (*BOLA*) system is the major histocompatibility complex (MHC) of cattle. The MHC genes, mapped to the bovine chromosome 23, play key roles in immune susceptibility and resistance to pathogens^48^. *PCK1* is a key enzyme for gluconeogenesis in bovine^49^ and in gene *PRSS2*, a substitution effect of the T allele of single nucleotide polymorphism (SNP) rs41256901 in protease was significantly associated with feed conversion ratio (FCR) and residual feed intake (RFI), and suggestively associated with DMI in beef cattle^50^.

### Key regulators

Regulatory impact factors, RIF1 and RIF2, were used to identify candidate TFs and COFs modulating the expression of DEGs. Based on those metrics, we identified 176, 185, and 214 TFs and (or) COFs for liver, muscle, and rumen tissues (Fig. 3), respectively (*P* value ≤ 0.05). Shared TFs and COFs were identified between liver and muscle (*n* = 13), muscle and rumen (*n* = 23), rumen and liver (*n* = 21) and liver, muscle, and rumen (*n* = 1, cofactor *BTK*). *BTK* is a key regulator of the B-cell receptor (*BCR*) signaling pathway^51^. This pathway plays a crucial role in regulating B-cell survival, proliferation, and maturation, and also regulates several downstream signaling pathways, including the MAPK and AKT pathways^52^. The full list of key regulators for each tissue is presented in Supplementary Files S7, S8 and S9 for liver, muscle, and rumen, respectively.

### Functional enrichment analysis

**Figure 4:**
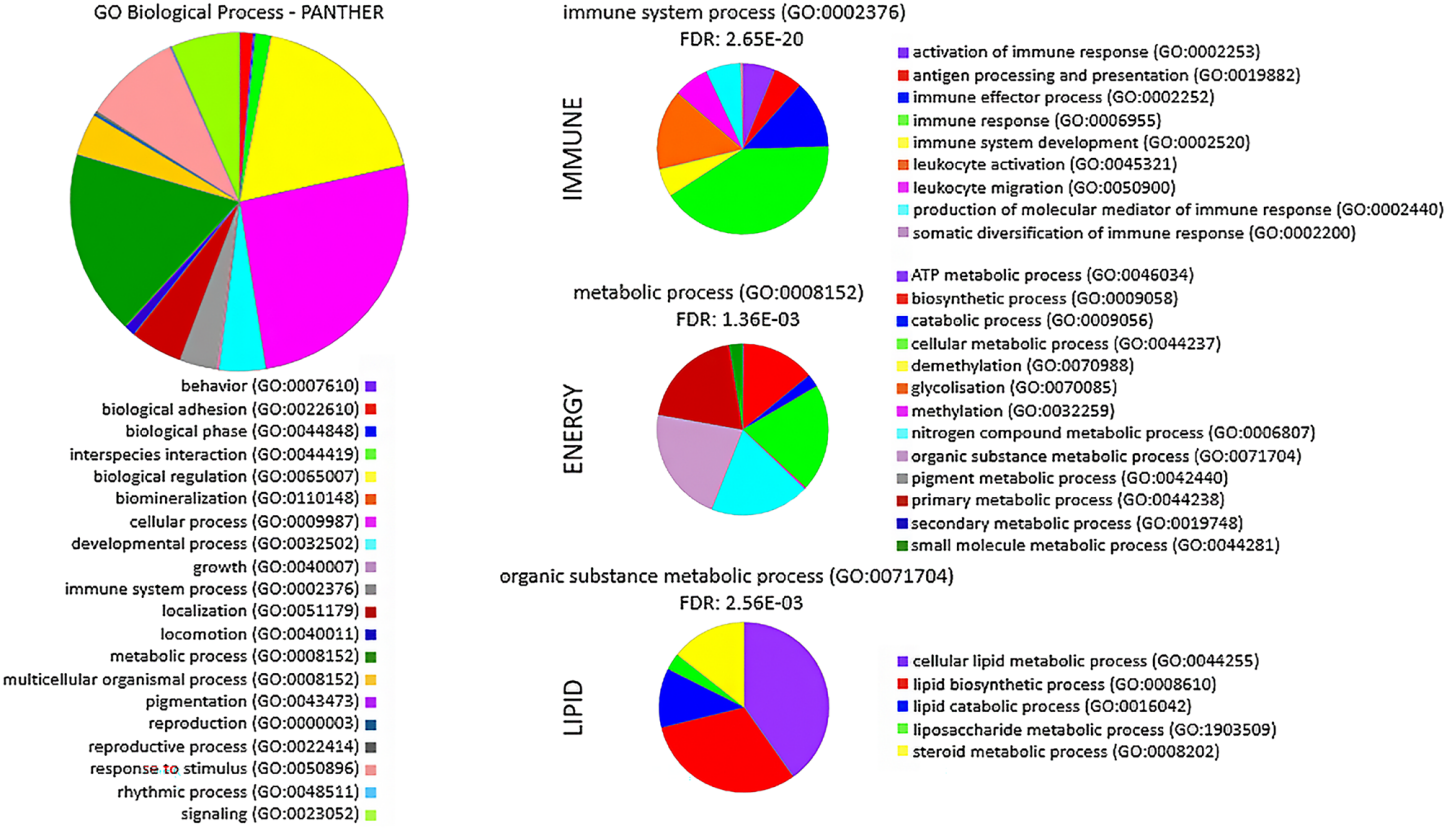
Pie charts representing the three key biological processes significantly enriched based on the list of differentially expressed genes and key regulators identified across all tissues, input for construction of the gene co-expression networks.

Based on the resultant list of DEGs, TFs and COFs identified across all tissues, and used as input for the construction of the gene co-expression networks, with the genes expressed in at least one tissue (14,776 genes) as a background list, a total of 464 gene ontology (GO) terms in the biological process (BP) category were significantly enriched (*P*-value < 0.05) (Supplementary File S10). In broad terms and across tissues, we observed enriched BPs such as immune function (FDR = 2.65 × 10^–20^), energy production (FDR = 1.36 × 10^–3^) and lipid metabolism (FDR = 2.56 × 10^–3^) (Fig. 4). Meanwhile the enriched tissue-specific BPs were indeed related to the tissues where they were preferentially expressed, that is regulation of primary metabolic process (FDR = 7.38 × 10^–8^) in liver; skeletal muscle tissue development (FDR = 5.02 × 10^–3^) in muscle and, regulation of inflammatory response (FDR = 3.28 × 10^–3^$$)$$ in rumen.

**Figure 5:**
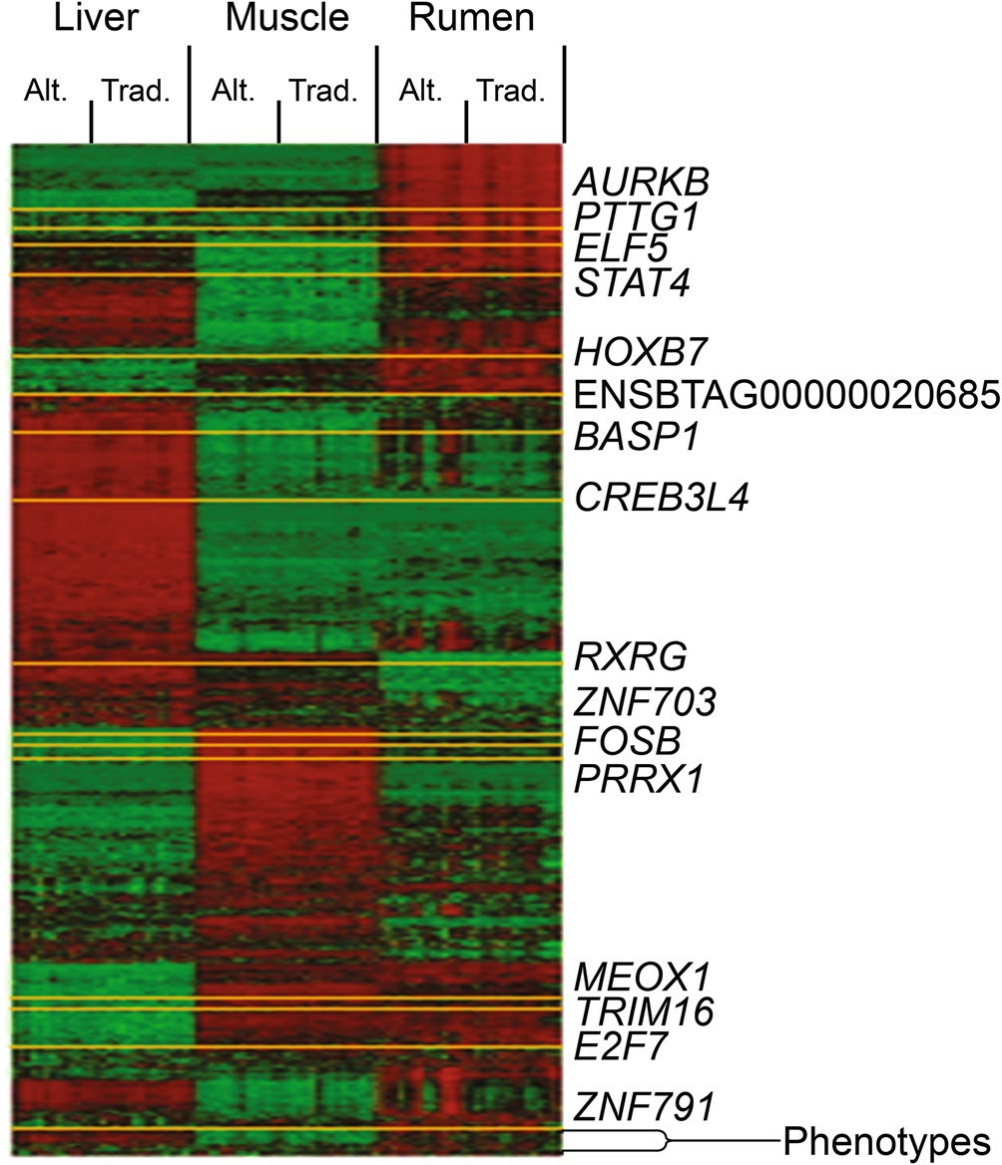
Heatmap of the hierarchical cluster analysis of differentially expressed genes, transcription factors and cofactors and the 14 phenotypes across the three tissues (liver, muscle and rumen) and two diets (Alt. = alternative diet; Trad. = traditional diet). Low and high expression are represented by green and red, respectively.

Furthermore, we performed functional overrepresentation analysis including only shared or unique genes identified across tissues. First, based on 235 shared genes among at least two out of the three tissues, a total of 205 BP GO terms were significantly enriched (Supplementary File S11). Similarly, based on tissue-specific genes, 60, 103 and 613 BP GO terms were significantly enriched in liver, muscle, and rumen, respectively (Supplementary File S12). As expected, the enriched BPs identified using shared and unique gene lists were comprised within the enriched BPs identified when analyzing all tissues together. Additionally, we constructed a hierarchical cluster heatmap pointing out the location of genes that became relevant after the differential expression and connectivity subsequent analyses (Fig. 5).

### Gene co-expression networks and differential connectivity

**Figure 6:**
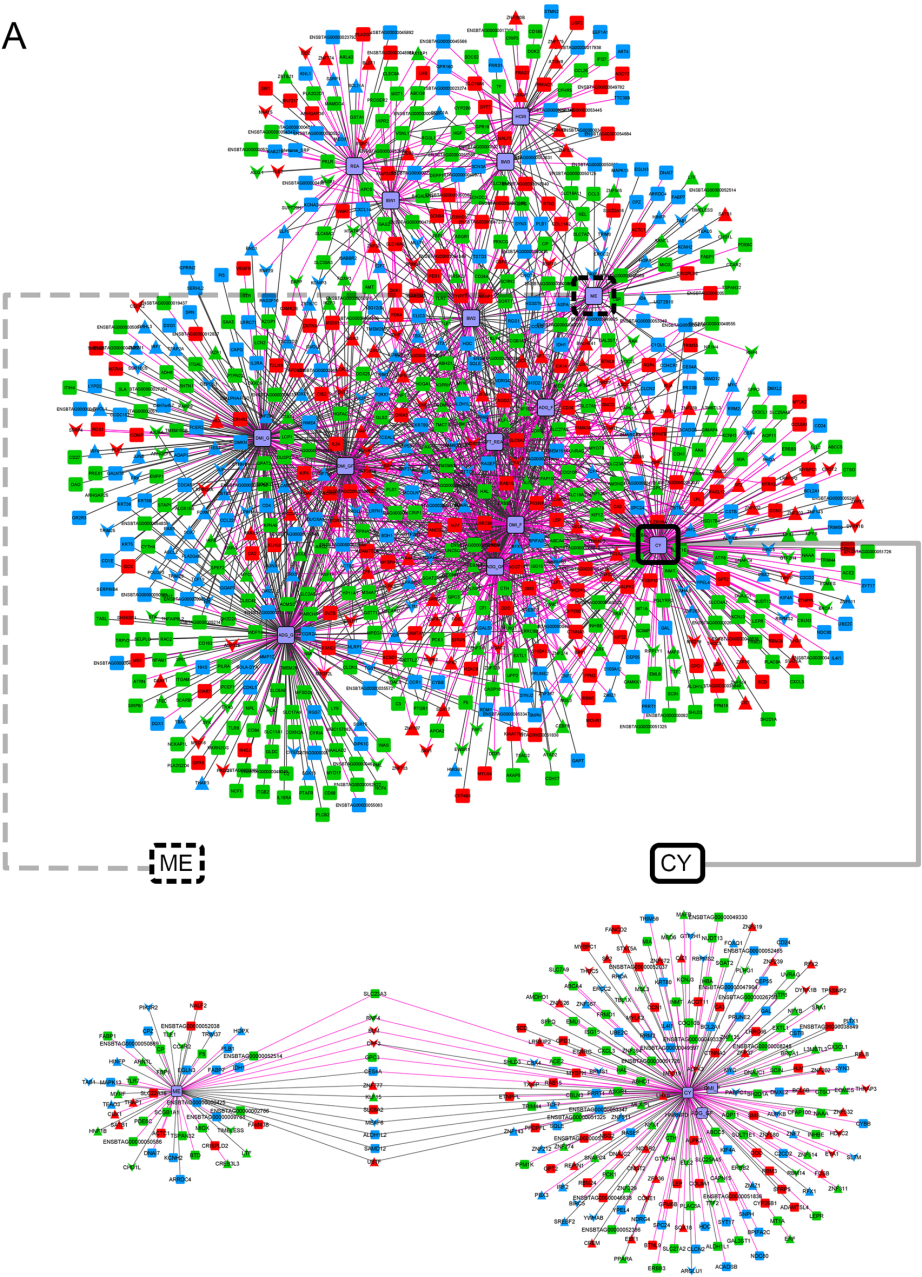

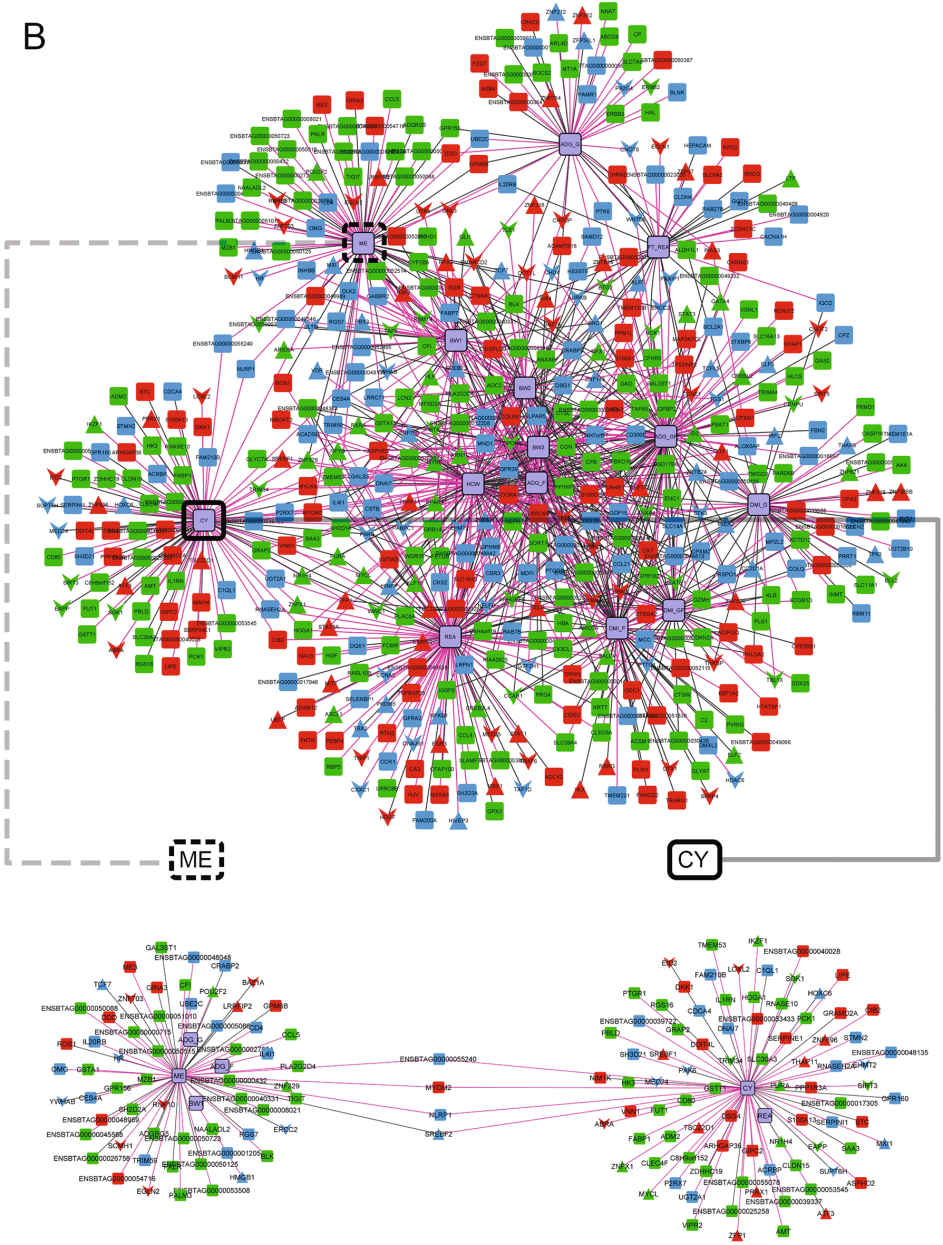
Gene co-expression networks with the unique significant connections for TRAD (**A**) and ALT (**B**) diets. Upper panels correspond to the visualization of the entire networks while the lower panels correspond to sub-networks, focusing on the phenotypes with the lowest and largest average changes in connectivity within diets and tissues (ME and CY, highlighted in dotted and solid black boxes, respectively). For the visualization schema, colours represent tissue of maximum expression: liver (green), muscle (red) and rumen (blue), with phenotypes represented in light purple with black borders; shapes represent genes or phenotypes (rectangle), transcription factors (triangle) or cofactors (inverted triangle); and, the colour of the edges represent type of correlation: positive (dark grey) or negative (pink).

Based on the PCIT algorithm, to infer the diet-specific networks involving all tissues we prioritized unique informative genes considering the following criteria: (1) DEG between TRAD and ALT diets; and (2) key regulators (TFs or COFs) based on RIF1 or RIF2 (Supplementary File S13). Thus, 1774 genes (DEGs and key regulators) + 14 phenotypes were used to construct the TRAD and ALT gene co-expression networks with significantly correlated pairs of nodes selected when at least one of the nodes was a phenotype. Based on that, the TRAD network contained 835 nodes and 1813 connections (implying a percentage of total possible connection or clustering coefficient of 0.52%), whereas the ALT network involved 525 nodes and 998 connections (clustering coefficient of 0.72%) (Fig. 6, Supplementary Files S14 and S15). Most TRAD network connections (*N* = 733, ~ 40%) belonged to muscle compared to 33% and 27% for liver (*N* = 595) and rumen (*N* = 485), while half of the ALT network connections (*N* = 499, 50%) belonged to liver compared to 31% and 19% for muscle (*N* = 309) and rumen (*N* = 190).

To explore differentially connected phenotypes across both networks, and under the assumption that gain or loss of connectivity in different biological situations would indicate changes in regulatory mechanisms, two sub-networks, one for each diet, were contrasted by focusing on the phenotypes with the largest change in average number of connections within diets and tissues. Specifically, methane emission had the lowest change in number of connections within the networks, while carcass yield had the highest change in connectivity between the two networks (Fig. 6, Table 6). In particular, among all connections, we highlight *SREBF2*, master regulator of sterol and fatty acid synthesis, which showed significant connections to ME and CY in the ALT network, however was only connected to CY (not to ME) and in liver (not in muscle) in the TRAD network.

**Table 6:** Number of connections by phenotype in each network, sorted by highest to lowest (last column).

Phenotypes^a^	Liver		Muscle		Rumen		Total
	ALT	TRAD	ALT	TRAD	ALT	TRAD	
CY	75	114	27	132	16	14	378
ADG_GF	76	74	31	99	22	29	331
DMI_F	26	80	25	116	35	31	313
DMI_GF	32	62	18	68	18	90	288
DMI_G	38	46	17	29	7	140	277
ADG_G	25	57	17	33	7	121	260
ADG_F	44	35	55	78	20	12	244
REA	77	31	31	31	36	21	227
BW2	38	55	45	54	15	12	219
HCW	63	26	34	26	21	13	183
FT_REA	15	34	20	66	5	26	166
BW3	43	21	28	31	15	13	151
BW1	29	21	29	32	12	18	141
ME	20	32	23	24	26	6	131

^a^For definition of phenotype abbreviation, see footnote on Table 2.

A closer examination of the number of connections for each phenotype in the six networks (Table 6) revealed that for CY the tissue with the largest change of connectivity between the ALT and the TRAD networks was muscle. Similarly, for ME the tissue with the largest change of connectivity between the ALT and the TRAD networks was rumen. This finding is of striking relevance because CY is highly influenced by muscularity while ME finds its origin in the rumen fermentation.

**Figure 7:**
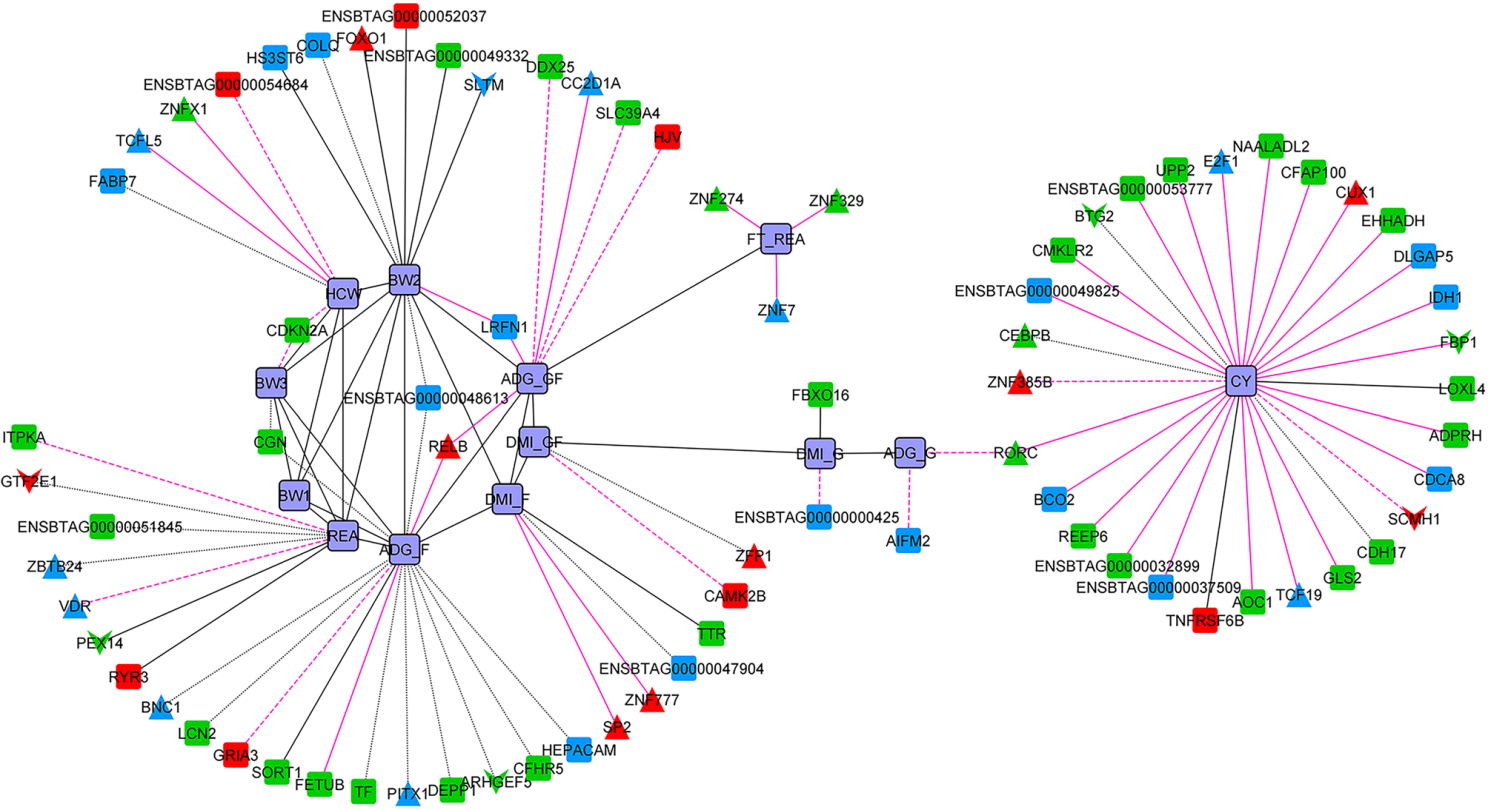
Gene co-expression network with the shared significant connections present in both diets. The line type represents the type of connection: solid (same signal in both networks), dash (negative in ALT and positive in TRAD) and dots (positive in ALT and negative in TRAD).

We also identified which significant connections were shared across the competing diets, creating a network with the connections present in both networks (Fig. 7, Supplementary File S16). The significant effect of diet on ME was further validated by the fact that no gene connections with ME were simultaneously observed in both the TRAD and ALT networks. In other words, the gene connections to ME were diet-specific. The attributes table we used to assist in the visualization and interpretation of the gene co-expression networks is presented in full in Supplementary File S17.

We determined which genes were differentially connected (DCGs) across diets, representing genes whose correlated expression pattern differed between both conditions. Note that all DCGs were also simultaneously DEGs, and genes with the highest change in the number of connections are likely to be key regulators^53^. We identified 162 DCGs between the TRAD and ALT networks (Supplementary File S18). The top 10 most DCGs were *SEMA7A*, *BASP1*, *NAAA*, *HNMT*, *TSPAN32*, *XAF1*, *RASGEF1A*, *CDCA5*, *ENSBTAG00000055140* and *AQP9.* Interestingly, all displayed maximum expression in rumen. Supplementary File S19 displays the CA-Plots comparing the number of connections of a gene against its average expression value, considering each tissue and diet comparison.

Lastly, we focused on the 17 TFs and COFs contained among the list of 162 genes that were differentially expressed and differentially connected (DEDC) (Table 7), highlighted as well in the hierarchical cluster heatmap (Fig. 5). The top 5 most differentially connected TFs or COFs across all tissues were *BASP1*, *ELF5*, *STAT4*, *PRRX1* and *RXRG*; all of them also displaying maximum expression in rumen. Within each tissue, the top ranked most differentially connected TF or COF were *MEOX1* (from 25 connections in the TRAD network to 100 in ALT), *PTTG1* (from 150 connections in the TRAD network to 18 in ALT) and *BASP1* (from 57 connections in the TRAD network to 366 in ALT) in liver, muscle, and rumen, respectively.

**Table 7:** DEDC TFs and COFs by diet and across tissues sorted by differential connectivity (Diff. Connec.)

Tissue	Gene symbol	Type	Expression		Connections		Diff. connec	*P* value	
			TRAD	ALT	TRAD	ALT	|TRAD − ALT|	DE	DC
Rumen	*BASP1*	COF	3.3903	4.0028	57	366	309	0.0016	0.0065
Rumen	*ELF5*	TF	3.3236	3.9761	271	57	214	0.0008	0.0093
Rumen	*STAT4*	TF	0.4694	1.0797	238	27	211	0.0017	0.0006
Rumen	*PRRX1*	TF	0.5084	1.0135	21	204	183	0.0075	0.0011
Rumen	*RXRG*	TF	− 0.5521	− 1.1625	176	30	146	0.0017	0.0041
Muscle	*PTTG1*	COF	0.1741	− 0.1766	150	18	132	0.0081	0.0002
Muscle	*AURKB*	COF	− 0.1184	− 0.5239	129	28	101	0.0027	0.0068
Muscle	*BASP1*	COF	3.1808	2.8104	97	13	84	0.0055	0.0005
Liver	*MEOX1*	TF	− 2.2663	− 1.7681	25	100	75	0.0007	0.0055
Liver	*ZNF703*	COF	0.673	1.0484	83	15	68	0.0075	0.0005
Liver	*ZNF791*	TF	− 0.1592	0.2076	5	70	65	0.0087	7.6 × 10^–7^
Liver	*E2F7*	TF	− 2.5227	− 2.0553	85	26	59	0.0013	0.0098
Liver	*HOXB7*	TF	− 2.0412	− 1.498	14	72	58	0.0003	0.0013
Liver	*FOSB*	TF	− 0.4205	0.0066	77	22	55	0.003	0.007
Rumen	*TRIM16*	COF	3.0366	2.5413	10	65	55	0.0086	0.0071
Muscle	*CREB3L4*	TF	− 0.3016	− 0.7554	9	59	50	0.0009	0.0001
Liver	ENSBTAG00000020685	TF	− 0.7306	− 0.2895	13	55	42	0.0023	0.0043

## Discussion

In terms of cost, feed accounts for a substantial proportion of production expenses in cattle farming^1^. Therefore, optimizing feeding strategies, such as feed formulation, considering nutrient composition, availability, and price, can be employed to improve efficiency, reduce costs, and enhance sustainability and profitability of cattle production. Exploring alternative feed ingredients, such as crop residues and industrial by-products, offers opportunity for cost savings, whilst also presenting potential means for reducing the environmental footprint associated with feed production^54^.

In this study, by integrating high-throughput RNA-seq data from liver, muscle and rumen—key tissues involved in metabolism, products synthesis, and nutrient digestion—with phenotypic information, we applied multi-tissue condition-specific co-expression network approaches, to identify changes in co-expression patterns of genes and phenotypes across competing diets, underlying diet-induced phenotypic variability. The incorporation of phenotypes as additional nodes in the network allowed us to uncover biologically relevant results, which are not evident by differential expression and co-expression analysis alone. In that regard, we reported significant differentially co-expressed genes and differentially connected phenotypes in young Nellore bulls. Furthermore, we revealed candidate TFs and COFs modulating the expression of DEGs across diets.

The transcriptional response to diet changes in metabolic tissues provides valuable insights into molecular mechanisms underpinning diet-induced variations in nutrient utilization, energy metabolism, and overall metabolic homeostasis^55^. The precise control of gene expression is orchestrated by diverse regulatory mechanisms, including TFs and COFs^56^. TFs and COFs play a critical role in shaping distinct gene expression patterns in accordance with nutrient availability^57^, acting as molecular switches that bind to specific DNA sequences and regulate the transcriptional activity of target genes, consequently influencing gene expression and ultimately leading to phenotype modulation^58^.

In terms of the phenotype level, at a *P* value < 0.05, ANOVA showed significant differences in favour of the alternative diet, including higher growth at finishing, lower methane emission, and higher carcass yield. The increase in growth suggests that the alternative diet may provide better nutritional support for growth compared to the traditional high-grain diet during the finishing stage of production. This finding is crucial in terms of livestock production efficiency and overall economic viability. Moreover, the lower methane emission associated with the alternative diet indicates that its composition might lead to improved rumen fermentation processes or reduced methane production per unit of feed consumed, which is particularly relevant in the context of environmental sustainability. This implies that the alternative diet may conduct to improved environmental outcomes compared to the traditional diet. Lastly, carcass yield is an important metric in meat production, reflecting the efficiency of converting feed into usable meat. The slight increase in carcass yield with the alternative diet advocates that it could promote better muscle development, leading to improved meat production efficiency, desirable from both economic and consumer perspectives. In summary, these findings demonstrate the potential benefits of the alternative diet over the traditional diet in terms of growth performance, feed efficiency, environmental impact, and potentially upgraded meat quality. However, further research is necessary to comprehensively understand the underlying mechanisms driving these effects, assess long-term implications, and evaluate the economic feasibility of implementing the alternative diet on farm on a larger scale.

At the transcriptional level, to provide an overview of the gene expression relationship across the tissues studied, we prioritized informative genes from the DEG, TF and COF analysis. We identified a set of genes that showed significant changes in expression levels across diet groups and tissues. The observed tissue-specific gene expression patterns suggested that each tissue responds uniquely to dietary factors, indicating the importance of considering multi-tissue transcriptomic analysis for a comprehensive understanding of the intricate relationship existent between diet and gene expression.

The functional enrichment analysis further elucidated the biological processes and pathways influenced by diet type. We identified enriched biological processes and pathways associated with specific diet-induced gene expression changes. These pathways included those involved in nutrient metabolism, energy utilization, immune response, and muscle development, among others. These pathways provide valuable insights into the underlying molecular mechanisms that mediate the observed phenotypic variations in beef cattle.

The gene co-expression analysis allowed us to identify diet-specific changes in gene co-expression patterns, one of the key findings from this study. We observed that certain genes showed altered co-expression relationships in response to different diets, indicating a rewiring of regulatory interactions in the presence of specific dietary factors. The incorporation of phenotypes in the gene network provided an enhanced ability to make inferences at the macro whole-of-body scale. Importantly, we observed significant correlations between diet-induced gene expression changes and phenotypic measures, with a focus on the phenotypes with the largest change in the average number of connections within diets and tissues, namely ME and CY. These changes in co-expression patterns suggest activation or suppression of specific biological processes and pathways in response to dietary interventions such as energy utilization and muscle development, ultimately impacting the phenotype.

As an adaptive response to changes in nutrient availability, the rewiring of key regulators is likely to influence the expression of target genes^57^. This was supported by the differential connectivity results that showed TFs and COFs differentially coordinating gene transcription between diets. In particular, we highlight as a pivotal revelation of our work, diet-induced gene expression changes involving *SREBF2*, a master transcription regulator for energy homeostasis and cholesterol biosynthesis^59^. In beef cattle, an association of cholesterol metabolism with feed efficiency has been reported by Karisa et al.^60^, and lower blood cholesterol content was observed in feed-efficient animals in comparison to inefficient counterparts^61,62^. In our study, *SREBF2* showed significant connections to ME and CY in the alternative diet network. Surprisingly, in the traditional network, *SREBF2* was only connected to CY (not to ME) and in liver (not in muscle), further suggesting differential phenotype regulation, in response to diet, through disruption/activation of biological pathways, eventually contributing to phenotype determination.

We identified *MEOX1*, *PTTG1*, and *BASP1* as the network nodes undergoing the most dramatic changes in connectivity, strongly suggesting key roles in mediating the effects of diet on gene expression and, consequently, phenotypic outcomes. *MEOX1* was previously identified in mice as a positive regulator of smooth muscle cells differentiation^63^, expressed during embryogenesis in the early developing somite^64^. Overexpression of *PTTG1* might affect the differentiation process of adipocytes^65^, and, through recruitment of cholesterol, *BASP1* may be involved with chromatin remodelling^66^, important for metabolic programming^67^. Their precise mechanism remains unknown.

Even though our study offers compelling insights, it is important to acknowledge its limitations. By recognizing such constraints, we can better contextualize and interpret the findings, ensuring a balanced perspective on the research outcomes. Firstly, the complexity of dietary factors and their interactions makes it challenging to attribute specific changes in co-expression patterns solely to a single dietary component. By nature, diet is a multifaceted entity comprising various nutrients and bioactive compounds, and their combined effects may contribute to the observed changes in gene co-expression. Further studies with controlled diet compositions are necessary to dissect individual contributions of specific nutrients. In addition, the specific diets used in this study may not fully represent the wide range of dietary variations encountered in commercial beef production systems. Future research incorporating diverse diet compositions and feeding regimens are warranted to capture the complexity of dietary effects on beef cattle phenotypes.

Moreover, the focus on liver, muscle, and rumen transcriptomes represents only a fraction of the complex molecular interactions occurring in beef cattle. Future investigation should explore other relevant tissues and molecular factors, such as epigenetic modifications and non-coding RNA, to gain a more comprehensive understanding of the diet-phenotype relationship. Finally, our study focused on a specific animal model and may not capture the full spectrum of diet-induced gene co-expression changes and phenotypic variations across different species or populations. Future studies can build upon these limitations to further advance our understanding of the subject matter.

To end on a practical note, for various operational reasons, including supply-chain availability, it might not be feasible to switch all cattle to the alternative diet. In that case, priority should be given to cattle with higher than average ME phenotypes and, among these, those exhibiting (1) high expression for *MYOM2* (in liver), *SCMH1* (in muscle) and *BLK* (in rumen) as the expression of these genes was the most negatively correlated with ME in the alternative diet network; and (2) low expression for *ZNF703* (in liver), *TRIM59* (in muscle) and ENSBTAG00000050869 (in rumen) as the expression of these genes was the most positively correlated with ME in the alternative diet network.

Similarly, this prioritization could be based on CY so that the alternative diet should first be given to cattle with lower than average CY, and among these, those exhibiting (1) low expression for *GSTT1* (in liver), *ARHGAP36* (in muscle) and *CD80* (in rumen) as the expression of these genes was the most negatively correlated with CY in the alternative diet network; and (2) high expression for *SGK1* (in liver), *DDIT4L* (in muscle) and *SUPT6H* (in rumen) as the expression of these genes was the most positively correlated with CY in the alternative network. For reference, even though validation of specific genes as reliable markers for dietary responsiveness require extensive research and validation efforts, as well as development of molecular assays and field trials, ongoing progress in the molecular biology field hold promise to transform our understanding of personalized nutrition. By validating candidate genes associated with desirable traits, we can streamline breeding programs to produce animals that thrive on specific diets, optimized for an individual’s genetic profile.

Lastly, as a final recommendation on how the alternative diet could be used to maximize its benefits on the farm, in light of the observed phenotypic variation analysis, it would be prudent for producers to consider strategically incorporating the alternative diet during the finishing stage to capitalize on its most significant effects. This targeted approach aligns with optimizing production efficiency while minimizing costs associated with feed formulation.

## Conclusions

In conclusion, the analysis of multi-tissue diet-specific gene co-expression networks enabled the identification of changes in gene co-expression patterns across diets, which in turn impacted phenotypic measures. Our study demonstrated that diet-specific gene co-expression networks can add important information into the regulatory mechanisms underpinning diet-induced phenotypic variability. The identification of diet-responsive differentially co-expressed genes and phenotypes offers a foundation for further investigations to expand upon our findings, ultimately conducting to the implementation of tailored precision nutrition approaches and improved beef production practices, leading to enhanced animal welfare, productivity, and sustainability in livestock farming. Taken together, we have comprehensively compared two competing diets at the macro (phenome) and at the micro (transcriptome) levels, showed the advantages of the by-product based alternative diet, and provided recommendations on how the transition to the alternative diet should be prioritized.

### Supplementary Information

Supplementary Information.

## Data Availability

The datasets used and/or analyzed during the current study were obtained under license from EMBRAPA and so cannot be publicly available. Data is however available from the corresponding author on reasonable request, and with authorization of EMBRAPA.

## Abbreviations

ADG_F: Average daily weight gain estimate relative to the finishing feedlot phase
ADG_G: Average daily weight gain estimate relative to the growing feedlot phase
ADG_GF: Average daily weight gain estimate relative to the growing + finishing feedlot phases
ALT: Alternative diet
ANOVA: Analysis of variance
BP: Biological process
BW1: Body weight taken at the beginning of the experiment
BW2: Body weight taken at the mid-point of the experiment
BW3: Body weight taken at the end of the experiment
COFs: Cofactors
CPM: Log2 counts per million
CY: Carcass yield
DCGs: Differentially connected genes
DEDCs: Differentially expressed and differentially connected genes
DEGs: Differentially expressed genes
DMI_F: Dry matter intake estimate relative to the finishing feedlot phase
DMI_G: Dry matter intake estimate relative to the growing feedlot phase
DMI_GF: Dry matter intake estimate relative to the growing + finishing feedlot phases
EMBRAPA: Brazilian Agricultural Research Corporation
FCR: Feed conversion ratio
FDR: False discovery rate
FT_REA: Fat thickness at the rib-eye muscle area
GO: Gene ontology
GS: Genomic selection
GWAS: Genome-wide association study
HCW: Hot carcass weight
LD: *Longissimus dorsi* Muscle
LSMean: Least-squared means
ME: Methane emission
PCIT: Partial correlation and information theory
PROC GLM: General linear model procedure
R^2^: Percentage of variation
REA: Rib-eye muscle area
RFI: Residual feed intake
RIF: Regulatory impact factor
RIN: RNA integrity number
SD: Standard deviation
SE: Standard errors
SNP: Single nucleotide polymorphism
TFs: Transcription factors
TMM: Trimmed mean of M-values
TRAD: Traditional diet
